# Size and Shape of Nanoclusters: Single-Shot Imaging Approach

**DOI:** 10.1002/smll.201102710

**Published:** 2012-05-29

**Authors:** Y Han, D S He, Y Liu, S Xie, T Tsukuda, Z Y Li

**Affiliations:** Nanoscale Physics Research Laboratory, School of Physics and Astronomy, University of BirminghamBirmingham, B15 2TT, UK E-mail: Z.Li@bham.ac.uk; Catalysis Research Center, Hokkaido UniversityNishi 10, Kita 21, Sapporo 001-0021, Japan

**Keywords:** gold clusters, cluster sizes, cluster shapes, stems

Gold nanoparticles on solid supports are of particular interest for their high catalytic activity for low-temperature CO oxidation.^1^ Although a number of factors influencing the catalytic properties have been recognized, including method of synthesis, size, shape, and interaction with support, the origin of the reactivity is still much of a debate.^2^ By way of example, Lopez et al. pointed out that, among these factors, the availability of a high concentration of low-coordination sites on small Au particles, particularly those at corners, is the most important.2b In contrast, Valden et al. showed that the catalytic rate of Au nanoparticles decreases with decreasing particle size when they are smaller than 3 nm, despite an increase in the amount of low-coordination atoms.^3^ In the later articles, the same group demonstrated that the catalytic activity of the bilayer Au films is the highest as compared with those of the bilayer and hemispherical Au particles.^4^ This further suggested that low-coordination corner or edge Au sites may not be so crucial as they were previously considered. More recently, based on the scanning transmission electron microscopy (STEM) analysis, a high catalytic activity for carbon monoxide oxidation is associated with the presence of bilayer Au nanoparticles of 0.5 nm on iron oxide.^5^ However, this is not supported in a subsequent article, in which the absolute necessity of monolayer or bilayer structure for high catalytic performance is ruled out.^6^ These distinct reports indicate that more in-depth research is needed in order to establish a clear link among these identified factors and any combination of them should be treated on a case-by-case basis.

Measuring size and shape of nanometer catalytic clusters is a challenging task. In particular, real catalyst samples are often characterized by a distribution of size and shape. Although the capability of STEM, with or without an aberration corrector, as a structural probe has been demonstrated,^7^–^11^ there have been few reports on viability of the technique as an effective tool for obtaining size and shape information of real nanocatalysts. Here, we apply this technique to size-selected model catalysts, Au_25_ and Au_39_ (clusters containing nominal 25 and 39 gold atoms, respectively). We show, by optimizing imaging conditions of a STEM equipped with a high-angle annular dark-field (HAADF) detector, that the size and shape of the clusters can be obtained from the STEM-HAADF intensity profile through single-shot imaging. The quantitative analysis of STEM-HAADF intensity allows us to derive that the deposited and then calcined Au clusters maintained discrete sizes being multiples of either Au_25_ or Au_39_. The overall shape of the clusters can be modelled as hemispherical-like, suggesting a strong cluster–support interaction.

**Figure** **1**a,b show two high-resolution images taken from samples of Au_25_ and Au_29_ supported on hydroxyapatite (HAP), respectively. Each cluster was analysed with the background subtracted with reference to its surrounding areas. The histograms of the integrated intensities for the samples Au_25_ and Au_39_ are shown in **Figure** **2**a,b, respectively. To determine the number of peaks (k) and the associated peak positions in each case, the Integrated Complete Likelihood (ICL) criterion was employed based on Gaussian distributions.^12^ The intensities larger than 120 × 10^4^ for Au_25_ and 95 × 10^4^ for Au_39_ were not included in the ICL calculation due to a small number of data points. The best fits were achieved when k = 8 for Au_25_ and k = 4 for Au_39_, corresponding the existence of 8 and 4 mixed Gaussian functions, respectively, as shown in Figure 2.

**Figure 1:**
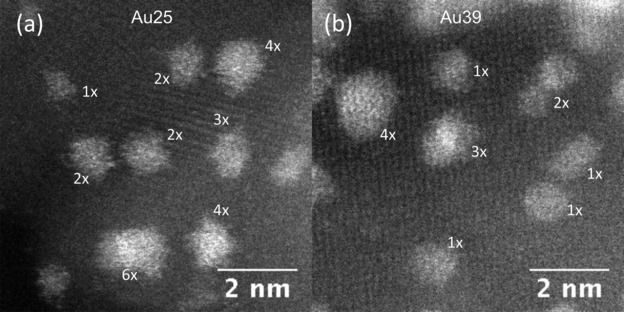
High resolution HAADF-STEM images taken from: a) Au_25_, and b) Au_39_ samples, respectively. The mass of the clusters were labeled as times of either Au_25_ or Au_39_ monometers in each case.

**Figure 2:**
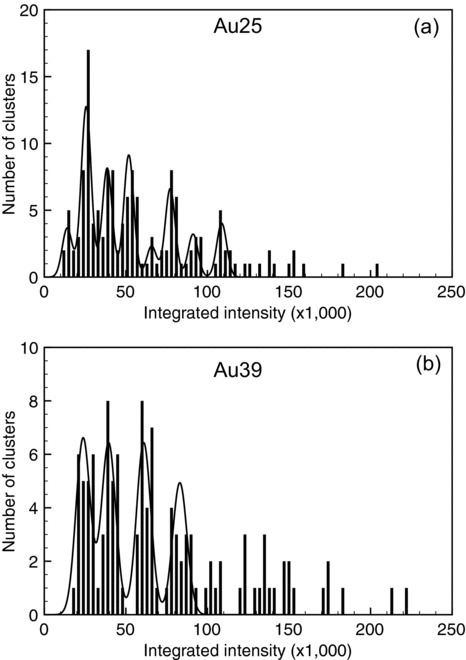
Histograms of integrated intensity and the corresponding Integrated Complete Likelihood (ICL) criteria evaluated as a function of the number of Gaussian functions for: a) the Au_25_, and b) the Au_39_ clusters on hydroxyapatite, respectively. The solid curves are the fitted Gaussian functions achieved from the ICL calculations.

There are several features of interest in Figure 2 that relate to the nature of the size-controlled Au clusters. The fitted Gaussian functions are evenly spaced in both cases. The spacing between the neighboring peaks for the Au_25_ sample is (136 ± 11) × 10^2^, with that for the Au_39_ sample being (208 ± 22) × 10^2^. The ratio of these two values is 1.53, being very close to the expected ratio of numbers of atoms in Au_39_ and Au_25_ monomers, i.e., 39/25 = 1.56. Therefore, the first peaks in each case are attributed to the corresponding monomers, with the rest of peaks being the multiples. The multiples of the Au_25_ and Au_39_ monomers are labeled in Figure 1 according to the above analysis.

Here, the size-controlled Au_25_ and Au_39_ nanoclusters were acting as mutual mass calibrators. In the quantitative analysis of the cluster intensities, a number of factors can introduce errors, including, for example, the emission current, the aberrations of the magnetic lenses, the position of the objective aperture to the Ronchigram, the position of the electron beam with regards to the ADF detector and the offset and gain values of the ADF preamplifier. In particular, to assess the microscopy conditions on quantification of mass of a cluster, two specimens, both containing single Au atoms supported on carbon films (images not shown in this paper), were used as an independent check between two different runs. The difference in the mean integrated intensity for a single atom is ∼5%, far smaller than the integrated intensity difference, ∼56%, between the Au_25_ and Au_39_ cluster samples. Whenever possible, both Au_25_ and Au_39_ samples were always imaged in the same session to compare.

The results in Figure 2 show that a large fraction of the clusters in both samples had aggregated, consisting several clusters of initial sizes. The clear integer multiples in the intensity histograms suggest that the aggregation is not via an Ostwald ripening process, rather it may be through cluster fusion, in which clusters retained their original identities. This is consistent with the broad cluster diameter distribution (full-width-half-maximum) in each case, 1.25 nm for Au_25_ and 1.5 nm for Au_39_, with the mean diameter of around 1.7–1.9 nm (See **Figure** **3**).

**Figure 3:**
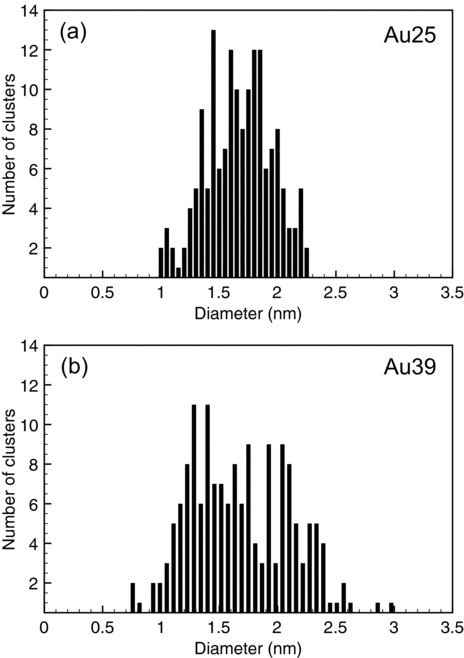
Histograms showing the diameters of the Au_25_ (a) and the Au_39_ (b) clusters on hydroxyapatite.

The above result should be compared with the previous published data for the same set of samples when they were imaged immediately after the synthesis, in which both types of the clusters had been shown to be 1.1 ± 0.5 nm in diameter.^13^ The large discrepancy in diameter distribution between the two studies cannot be completely attributed to the limited resolution and low sensitivity of the STEM without a probe corrector used in the previous study. One possibility is that the aggregation had occurred during the time span over two months from the sample preparation in Japan to the STEM observations in the UK. Further investigation is planned to see if these aggregated clusters, which have retained the memory of their initial sizes, still maintain their size-dependent catalytic performance.

To gain insight of the bonding strength between clusters and the HAP support, we plot in **Figure** **4** the integrated HAADF intensity of each cluster as a function of its diameter, together with the relationship generated from the idealized spherical and hemispherical models (shown with solid and dashed lines). In the geometric models, the Wigner-Seitz radius of gold, *r*_ws_ = 0.165 nm, is used.^10^, ^14^ It is clear from Figure 4 that majority data points, though scattered, follow the trend of the hemispherical model, when the diameter is less than ∼2 nm. The result suggests that the clusters are flattened on the support, likely as a result of the strong interaction between the Au and PO_4_^3−^ moieties of HAP.^15^

**Figure 4:**
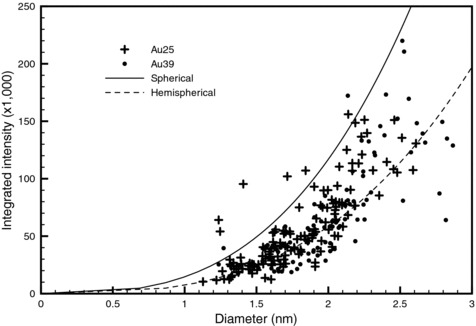
Integrated HAADF intensity of clusters as a function of corresponding diameter. The solid and dashed lines represent idealized spherical and hemispherical models, respectively.

In summary, Au_25_ and Au_39_ nanoclusters supported on hydroxyapatite (HAP) were investigated using aberration corrected scanning transmission electron microscopy. We demonstrate that the quantitative analysis of the HAADF intensities of the nanoclusters provide an invaluable tool in obtaining direct information of cluster size in terms of the number of atoms within. We show that the well size-controlled clusters, as in this work, can act as ideal mass calibrators, similar as those prepared in the gas phase and mass selected.^9^, ^10^, ^16^ We have associated the evenly spaced peaks in the histograms with multiples of single clusters of either Au_25_ or Au_39_ in each case, from which Oswald ripening mechanism for the observed cluster aggregation can be ruled out. There is a clear sign indicating that the aggregated clusters retained their original identity. This feature together with the strong interaction with the hydroxyapatite support based on the 3D shape analysis suggests new opportunities in the development of stable Au catalysts with size-dependent reactivity.

## Experimental Section

The synthetic method of glutatoneate-protected Au (Au:SG) clusters has been reported previously.^13^, ^17^ Briefly, chilled (0 °C) aqueous solution of NaBH_4_ was rapidly injected into the methanol solution of glutathione (GSH) and HAuCl_4_ kept at 0 °C under vigorous stirring. The precipitates were collected and dried in vacuo to obtain crude mixture of Au:SG. The Au_25_(SG)_18_ and Au_39_(SG)_24_ clusters were isolated using polyacrylamide gel electrophoresis; the compositions and purities were confirmed by electrospray ionization mass spectrometry.

The Au_*n*_ clusters (*n* = 25, 39) supported on hydroxyapatite (HAP), Ca_10_(PO_4_)_6_(OH)_2_, were prepared via two steps.^13^, ^18^ Firstly, the Au_25_(SG)_18_ or Au_39_(SG)_24_ clusters were adsorbed on HAP (0.2 wt%) by mixing them with HAP in basic water. Secondly, the Au_n_(SG)_m_/HAP composites collected by filtering were calcined at 300 °C for 2 hours in vacuo. The GS ligands were confirmed to have been removed in this step, as evidenced by inductively coupled plasma analysis.

The Au_25_ and Au_39_ clusters supported on HAP were dispersed in methanol and loaded onto holey carbon films on copper grids directly. They were characterized using a JEOL 2100F transmission electron microscope, equipped with a CEOS probe aberration corrector, operated at 200 kV. The semi-convergence angle for the incident electron beam was ∼20 mrad and the inner and an outer collection angles for the JEOL ADF detector were 55 mrad and 148 mrad, respectively. The same values for the gain and offset of the preamplifier were used throughout the observations of the two samples. The emission current was monitored to be stable. In order to minimize the effect of the electron beam to both the clusters and the HAP substrate, the experimental conditions were carefully tuned, with a 20 μm condenser aperture, a medium magnification (×10M) and a short exposure time (20 μs/pixel) employed.
